# Spray-Formed Layered Polymer Microneedles for Controlled Biphasic Drug Delivery

**DOI:** 10.3390/polym11020369

**Published:** 2019-02-20

**Authors:** Seok Chan Park, Min Jung Kim, Seung-Ki Baek, Jung-Hwan Park, Seong-O Choi

**Affiliations:** 1 Nanotechnology Innovation Center of Kansas State, Kansas State University, Manhattan, KS 66506, USA; schpark@ksu.edu (S.C.P.); mj1217@ksu.edu (M.J.K.); 2 Department of Anatomy and Physiology, College of Veterinary Medicine, Kansas State University, Manhattan, KS 66506, USA; 3 QuadMedicine R&D Centre, QuadMedicine Inc., Seongnam 13209, Korea; bsk@quadmedicine.com; 4 Department of BioNano Technology, College of BioNano Technology, Gachon University, Seongnam 13120, Korea; pa90201@gachon.ac.kr; 5 Gachon BioNano Research Institute, Gachon University, Seongnam 13120, Korea

**Keywords:** layered microneedles, heterogeneous composition, biphasic release, rapid drug release, sustained drug release, spray deposition

## Abstract

In this study we present polymeric microneedles composed of multiple layers to control drug release kinetics. Layered microneedles were fabricated by spraying poly(lactic-*co*-glycolic acid) (PLGA) and polyvinylpyrrolidone (PVP) in sequence, and were characterized by mechanical testing and ex vivo skin insertion tests. The compression test demonstrated that no noticeable layer separation occurred, indicating good adhesion between PLGA and PVP layers. Histological examination confirmed that the microneedles were successfully inserted into the skin and indicated biphasic release of dyes incorporated within microneedle matrices. Structural changes of a model protein drug, bovine serum albumin (BSA), in PLGA and PVP matrices were examined by circular dichroism (CD) and fluorescence spectroscopy. The results showed that the tertiary structure of BSA was well maintained in both PLGA and PVP layers while the secondary structures were slightly changed during microneedle fabrication. In vitro release studies showed that over 60% of BSA in the PLGA layer was released within 1 h, followed by continuous slow release over the course of the experiments (7 days), while BSA in the PVP layer was completely released within 0.5 h. The initial burst of BSA from PLGA was further controlled by depositing a blank PLGA layer prior to forming the PLGA layer containing BSA. The blank PLGA layer acted as a diffusion barrier, resulting in a reduced initial burst. The formation of the PLGA diffusion barrier was visualized using confocal microscopy. Our results suggest that the spray-formed multilayer microneedles could be an attractive transdermal drug delivery system that is capable of modulating a drug release profile.

## 1. Introduction

Microneedles are designed to deliver pharmaceutical compounds across the skin in a minimally invasive manner and could offer a number of advantages over traditional needle injections, including reduced injection pain and improved patient compliance [1,2,3]. Since microneedles can target the antigen-presenting immune cells of the skin, application of microneedles in vaccination and immunotherapy has received great attention in recent years [4,5]. When composed of biocompatible polymers, microneedles can be manufactured at low costs and be safely disposed of after use [6,7]. Additionally, polymeric microneedles can be designed to achieve a desirable drug release profile by utilizing the dissolution and degradation properties of materials. For example, rapid drug release can be obtained by constructing microneedles from polymers that dissolve quickly in water, and has been demonstrated using various drugs and vaccines [8,9,10,11,12,13]. In some cases, sustained release of drugs is desirable to improve the treatment efficiency and patient compliance and to reduce undesirable side effects by providing optimal drug concentrations over a prolonged period [14,15]. In order to achieve prolonged drug delivery, poly(lactic-*co*-glycolic acid) (PLGA) has been widely studied to form microneedles [16,17,18,19,20]. For some drugs or vaccines, however, biphasic release would be more desirable to maximize therapeutic efficiency and reduce administration frequency. For example, single immunization could be feasible with the combination of rapid and sustained releases of antigens. It has been reported that the rapid antigen release initiates the immune response, and the following sustained release helps induce the protective level of immunity [21,22,23]. Furthermore, biphasic delivery of therapeutic proteins, such as growth factors and hormones, can be highly effective because the rapid release followed by the sustained release maintains the concentration of the protein within the therapeutic window for an extended period [24,25]. The controlled delivery over a sufficiently long duration would afford the maximized therapeutic efficiency and reduce injection frequency, thereby increasing patient comfort and compliance.

**Figure 1:**
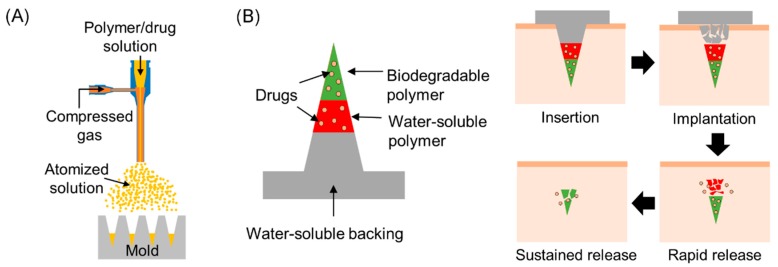
(**A**) Schematic diagram of spray deposition setup for polymer microneedle fabrication. (**B**) The concept of layered microneedles for biphasic drug delivery. Once inserted, the microneedle would be embedded in the skin due to dissolution of water-soluble polymers, and rapidly release drugs incorporated in a water-soluble matrix. Biodegradable tips would remain in the skin, releasing drugs for an extended period of time.

This work presents layered microneedles that enable biphasic release of drugs, which could be useful to fulfill the aforementioned therapeutic needs, and proposes a simple strategy to modulate release profiles. Previously, we demonstrated that microneedles could be formed by spray deposition [26], and that the spray-formed microneedles were capable of retaining the stability of proteins [27]. Based on our previous work, we further investigated in vitro drug release profiles of layered microneedles and attempted to control the release profiles by forming a diffusion barrier layer in the microneedles. The spray deposition setup shown in Figure 1A was used to fabricate microneedles from biodegradable PLGA and water-soluble polyvinylpyrrolidone (PVP). As illustrated in Figure 1B, we envisioned that the drug-containing part of microneedles would be fully embedded in the skin upon insertion by the dissolution of the bottom part of microneedles, and drugs in the PVP layer would be released immediately while drugs in the PLGA layer would be released slowly for a prolonged time period, thereby achieving biphasic release. In addition to release profiles, we also present the mechanical stability and skin penetration capability of layered microneedles, drug loading capacity, and the structural stability of bovine serum albumin (BSA) incorporated in PLGA and PVP.

## 2. Materials and Methods

### 2.1. Materials

Polyvinylpyrrolidone (PVP, MW 10 and 40 kDa), sulforhodamine B (SRB), coumarin 314, bovine serum albumin (BSA), and fluorescein isothiocyanate-labelled BSA (FITC-BSA) were purchased from Sigma-Aldrich (St. Louis, MO, USA). Poly(dl-lactide-*co*-glycolide) (50:50 PLGA, ester-terminated, inherent viscosity 0.26–0.54 dL/g) was purchased from Lactel Absorbable Polymers (Birmingham, AL, USA). Ethyl acetate (American Chemical Society (ACS) Reagent Grade), dimethyl carbonate (anhydrous, 99%), and glycerol (ACS Reagent Grade) were purchased from Fisher Scientific (Waltham, MA, USA). Polydimethylsiloxane (PDMS, Sylgard 184) was purchased from Dow Corning (Midland, MI, USA).

### 2.2. Fabrication of Layered Microneedles Using a Spray Deposition Process

A commercial coaxial needle (Ramé-Hart Instrument Co., Succasunna, NJ, USA) was used as a spray nozzle. The outer needle (15 gauge) was connected to a compressed air source, and a polymer solution was fed through the inner needle (21 gauge) by a syringe pump (AL-1000, World Precision Instrument, Sarasota, FL, USA). Microneedle molds were prepared by casting PDMS onto a microneedle array master that contained 100 pyramidal-shaped microneedles (300 × 300 × 600 µm, W × D × H). In order to form PLGA and PVP layers, polymer solutions (1% *w*/*v* PLGA in a mixture of ethyl acetate and dimethyl carbonate (1:4 *v*/*v*) and 5% *w*/*v* PVP (MW 10 kDa) in deionized water) were sequentially sprayed into the microneedle mold with 15 min drying between depositions. The detailed spray parameters for each polymer solution are described in our previous paper [26]. SRB and coumarin 314 were added to the aqueous PVP and organic PLGA solutions, respectively, for visualization. After spray depositions, a solution composed of 40% *w*/*v* PVP (MW 40 kDa) and 2.5% *v/v* glycerol in deionized water was cast to form a backing layer and dried overnight at room temperature. Upon separation from the mold, the fabricated layered microneedles were examined under an inverted fluorescence microscope (Olympus IX-73, Tokyo, Japan) coupled with a digital camera and integrated software.

### 2.3. Quantification of Proteins Encapsulated in PLGA–PVP Layered Microneedles

Two sets of PLGA–PVP microneedles, which contain a drug in only one of the layers, were prepared to determine the total amount of drug loaded in each layer. BSA was used as a model protein drug and was added to the aforementioned PVP and PLGA spraying solutions by direct mixing and emulsification, respectively. Water-insoluble urethane resin (Alumilite White, Alumilite Co., Kalamazoo, MI, USA) was used as the microneedle backing material to minimize assay interference caused by the dissolution of the backing. The final concentration of BSA in the PVP solution was 0.5% *w*/*v*, and the BSA–PLGA solution was prepared by emulsifying 2% *w/v* aqueous BSA solution containing 2% *w*/*v* PVA within the PLGA solution at the aqueous-to-organic phase volume ratios of 1:15 and 1:20. Layered microneedles containing different amount of BSA were prepared by changing the number of depositions (1, 2, 3, and 6 times). BSA in the PVP layer was extracted by immersing the fabricated microneedle in 1 mL of phosphate-buffered saline (PBS) for 30 min. The preliminary experiment found that there was no interference of PVP up to 2000 µg/mL (data not shown), and the influence of PVP could be neglected in the quantitative assay as the maximum concentration of PVP was approximately 800 µg/mL when the PVP solution was deposited 6 times. To extract BSA from the PLGA layer, the PLGA layer was selectively dissolved in 2 mL of acetone for 10 min, and the precipitated BSA in acetone was collected by centrifugation (25,800× *g* for 10 min). The collected BSA pellet was then reconstituted in 1 mL of PBS for quantification. The amount of BSA in each layer was quantified by the Bradford protein assay (Pierce™ Coomassie (Bradford) Protein Assay Kit, Thermo Fisher Scientific, Waltham, MA, USA) according to the manufacturer’s protocol. A total of 12 microneedles per group were quantified, and data were displayed as mean ± standard deviation. 

### 2.4. Skin Insertion Tests

Insertion of the layered microneedles into skin was examined using porcine cadaver skin obtained from the necropsy facility at Kansas State University according to the University’s Animal Use Guidelines. After removing subcutaneous fat and hair, 10 × 10 PLGA–PVP layered microneedle arrays were inserted into the pig skin using a thumb force (approximately 3–5 N) [28] and removed after 1 min. A blue tissue marking dye (Mark-It^TM^, Richard-Allan Scientific Co., San Diego, CA, USA) was applied to the skin for 1 min to selectively stain the microneedle insertion sites. After wiping residual dye from the skin surface with dry tissue paper, the skin was observed under a microscope (Olympus IX-73, Tokyo, Japan). To examine the cross-section of the insertion sites microscopically, the skin samples were embedded in optimal cutting temperature (OCT) compound (Tissue-Tek, Sakura Finetek USA, Torrance, CA, USA), snap-frozen in anhydrous 2-methylbutane, and cut into 10 µm thick transverse sections.

### 2.5. Mechanical Testing of Microneedles

The mechanical behavior of the layered microneedles was examined by a universal static testing system (5943 single column materials testing system, Instron, Norwood, MA, USA). The fabricated microneedles were affixed to the lower compression platen using double-sided tape and pressed by the upper platen at a speed of 1.3 mm/min according to ASTM D695-15 [29]. Five 3 × 3 arrays were subjected to the test. For comparison, single-layer microneedles (i.e., PVP or PLGA microneedles) with the same geometry were also tested. Data were collected until the displacement reached 400 µm, and the averaged data with standard deviation were displayed as force-per-needle versus displacement. Deformation of microneedles after the application of different compressive forces was examined by scanning electron microscopy (SEM).

### 2.6. Measurement of Conformational Changes in Proteins

To examine the changes in protein structure caused by the spray deposition process, the secondary and tertiary structures of BSA encapsulated in PLGA and PVP layers were measured as previously described [27]. Briefly, BSA-containing PLGA and PVP solutions were sprayed onto a PDMS substrate using the same procedures as for the microneedle fabrication. After drying, BSA in polymer matrices was extracted, resuspended in deionized water at a concentration of 200 μg/mL, and measured by the Bradford protein assay. The secondary structure of BSA was examined by circular dichroism (CD) spectroscopy (Jasco J-810, Tokyo, Japan) in the far-UV region (200–260 nm). The obtained CD spectra were analyzed using the Dichroweb [30,31,32] CDSSTR program [33]. Changes in the tertiary structure of BSA were evaluated by measuring the intrinsic fluorescence of BSA at an excitation wavelength of 280 nm using a microplate reader (Synergy H1, BioTek, Winooski, VT, USA). For all the experiments, native BSA and acid-treated BSA (pH 3 with 0.1 M hydrochloric acid) were used as controls. Six samples were prepared for each condition, and triplicate measurements were performed for each sample. Data were compared using a Student’s *t* test with equal variances. All data were presented as mean ± standard deviation. A value of *p* < 0.05 was considered statistically significant.

### 2.7. In Vitro BSA Release Study

In vitro release profiles of BSA from each layer were studied using the layered microneedles that contained BSA in either PVP or PLGA. Water-insoluble urethane resin was used as the microneedle backing to minimize interference during the release study. PLGA would cause insignificant interference during the release study due to its water insoluble nature and slow degradation rates (approximately 1–2 months) [34]. To obtain sufficient sample amounts for assay, three microneedle patches were immersed in 2 mL of PBS at 32 °C. At predetermined time intervals, 1 mL of the sample solution was withdrawn and the withdrawn sample volumes were replaced by fresh PBS. The amount of BSA released in PBS was determined by the Bradford protein assay, and the cumulative percentage release of BSA was calculated. The experiment was repeated six times for each condition.

### 2.8. Examination of the Deposition Profile of the Blank PLGA Layer Using Confocal Microscopy

To form a PLGA diffusion barrier layer, 1% *w*/*v* PLGA solution in a mixture of ethyl acetate and dimethyl carbonate (1: 4 (*v*/*v*)) was sprayed one or three times onto a microneedle mold using the following spraying parameters: flow rate of 1 mL/min, gas pressure of 35 kPa, spaying distance of 5 cm, and spraying duration of 3 s. Coumarin 314 (green fluorescent dye) was added to the PLGA solution for clear visualization. After the spray deposition, the microneedle mold was dried for 15 min at room temperature and was cut into approximately 1 mm pieces vertically to examine the cross-sectional view of the mold cavity. The z-stack images of the samples were acquired using a confocal microscope (Zeiss LSM 700, Carl Zeiss AG, Oberkochen, Germany), and the obtained images were reconstructed as a 3D image using digital imaging software (ZEN 2, Carl Zeiss AG).

## 3. Results and Discussion

### 3.1. Fabrication of Layered PLGA–PVP Microneedles

**Figure 2:**
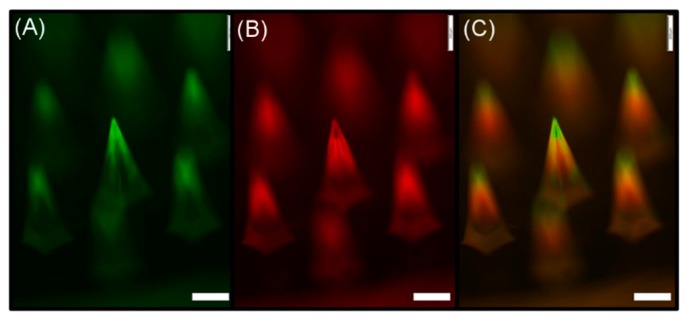
Fluorescence microscopy images of the fabricated layered microneedles. (**A**) Coumarin 314 (green)-loaded poly(lactic-*co*-glycolic acid) (PLGA) layer, (**B**) sulforhodamine B (SRB) (red)-loaded polyvinylpyrrolidone (PVP) layer, and (**C**) overlay of images (**A**,**B**). Scale bars represent 200 μm.

PLGA–PVP layered microneedles were fabricated by sequential deposition of PLGA and PVP solutions into PDMS microneedle molds, as shown in Figure 2. Coumarin 314 (green) and SRB (red) fluorescent dyes were added to PLGA and PVP solutions, respectively, to distinguish each layer. It was observed that PLGA was localized at the top portion of the microneedles, with a trace of green fluorescence along the sharp edges of the microneedles. The PVP layer indicated by red fluorescence, however, was formed below the PLGA layer without significant deposition of materials along the edges. This result indicates that the deposition profile of materials depends on the wettability of a spraying solution on the mold material, and can be controlled by manipulating interactions between the polymer solution and mold surface. For example, undesired deposition on the edge could be minimized by increasing the contact angle of polymer solutions on the mold through the chemical or physical modification of the mold surface.

**Figure 3:**
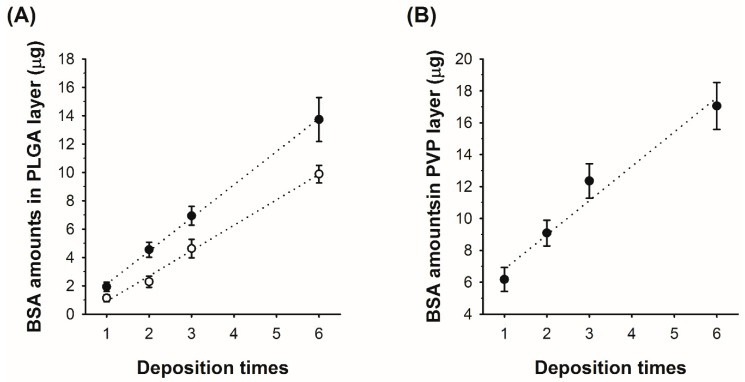
Total amount of bovine serum albumin (BSA) loaded in each layer of the microneedles according to the number of spray depositions. (**A**) The amount of BSA in the PLGA layer. Open and filled circles represent 1:20 and 1:15 volume ratios of aqueous BSA to organic PLGA solutions, respectively. (**B**) The amount of BSA amounts in the PVP layer.

After verifying the formation of PLGA–PVP layers in the microneedle, we further studied the relationship between the number of spray depositions and the amount of BSA in each layer. For PLGA, we also examined the deposited protein amount in relation to initial BSA concentrations in the PLGA solution. For both PVP and PLGA, the amount of BSA in each layer increases linearly with the increase of the number of depositions, as shown in Figure 3. When the PLGA/BSA emulsions with 15:1 and 20:1 *v*/*v* ratios were used for spraying, BSA deposition rates were approximately 2.3 μg and 1.7 μg per spraying with R-squared values of 0.994 and 0.992, respectively (Figure 3A). For the PVP/BSA aqueous solution (5% PVP and 0.5% BSA *w*/*v*), the amount of BSA deposited into the mold increased from 6.18 μg (single deposition) to 17.05 μg (six depositions) with an R-squared value of 0.963 (Figure 3B). The results demonstrate that drug loading amounts can be controlled by adjusting spraying parameters and can be estimated at a given initial drug concentration.

### 3.2. Influence of Spray Deposition on the Integrity of the Model Protein

Since conformational changes in protein structure could alter protein functions, it is highly important to minimally affect the three-dimensional structures of proteins during fabrication processes. To examine the effect of the spray deposition process on the protein structure, the integrity of secondary and tertiary structures of BSA was assessed by CD and fluorescence spectroscopy as previously described [27]. BSA was chosen as a model protein because BSA is easily accessible, well characterized, and has been widely used for studying protein stability in drug delivery systems [35,36,37]. Table 1 summarizes the conformational changes of BSA incorporated in PLGA and PVP layers, and the corresponding CD and fluorescence spectra are shown in Figure S1A,B, respectively. Native and acid-degraded BSA samples were used as controls for comparison.

When treated with acid, BSA underwent significant conformational changes (i.e., decreased α-helix content and intrinsic fluorescence intensity), which is consistent with prior literature [38,39]. For BSA extracted from PLGA and PVP layers, both secondary and tertiary structures were slightly disturbed during the spray deposition process. Although there were no significant differences in α-helix content, fluorescence data suggested that the three-dimensional shape of BSA encapsulated in PLGA was more impaired than that of BSA in PVP. Indeed, the tertiary structure of the BSA in the PVP layer was almost intact compared with native BSA. These results indicate that emulsification, which was used for preparing a BSA-containing PLGA solution, would contribute more to the conformational change of BSA than the mechanical stress exerted on BSA upon atomization in the spraying process because the spraying pressure used for fabricating the BSA–PVP layer (172 kPa) was much higher than that used for the BSA–PLGA layer formation (35 kPa). We anticipate that the addition of proper protein stabilizers, such as sugars and surfactants, to the spraying solution would further improve the structural stability of proteins by reducing the interfacial stress occurring during the spray deposition process. In order to further study the impact of the spraying process on protein stability, however, it is highly recommended to use labile proteins, since BSA could recover its native structures to some extent when placed in a proper buffer solution [40,41].

### 3.3. Mechanical Testing of Microneedles

**Figure 4:**
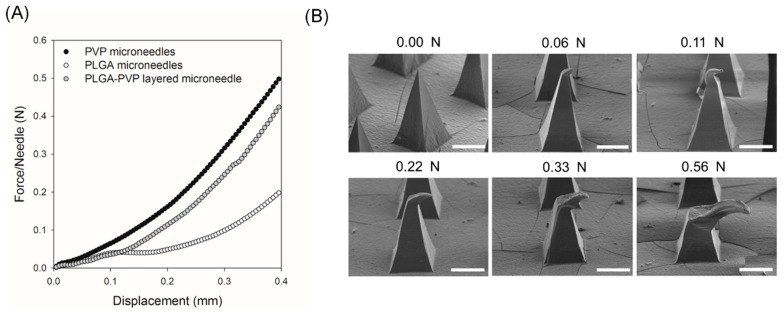
Mechanical behavior of PLGA–PVP layered microneedles. (**A**) Force–displacement curves generated by a compression test. (**B**) SEM images of the layered microneedles after applying different compressive forces. Scale bars represent 200 μm.

The mechanical behavior of PLGA–PVP-layered microneedles under uniaxial compression was examined using a mechanical tester and compared with monolithic microneedles (i.e., PLGA-only and PVP-only microneedles). As shown in Figure 4A, the force–displacement curve of PLGA–PVP microneedles followed that of PLGA microneedles until the displacement reached approximately 0.12 mm, which corresponds well to the length of the PLGA tips. Thereafter, the slope of the curve was almost identical to that of PVP-only microneedles, indicating the compression of the PVP portion of PLGA–PVP microneedles. During the test, we did not observe any sharp drop in the force, suggesting that there was no delamination or buckling occurring at the PLGA–PVP interface. In order to further evaluate the adhesion between PLGA and PVP layers, the shape of the layered microneedles under different compressive loads was observed by SEM (Figure 4B). Although the structure was severely deformed by compression, no crack was identified at the PLGA–PVP interface, confirming that reliable adhesion between two layers was achieved. We anticipate that the adhesive properties of PVP [42,43] contributed to the structural integrity, which is highly important to ensure the insertion of microneedles without breakage.

### 3.4. Insertion of Microneedles into Porcine Cadaver Skin

The PLGA–PVP layered microneedles were aimed to be used as implantable drug delivery devices that are capable of modulating drug release. To achieve this goal, microneedles should be able to penetrate the skin and demonstrate drug release at different rates. Thus, we examined the skin penetration and drug delivery ability of the multilayer microneedles using porcine cadaver skin. The fabricated microneedles were gently pressed against the skin by the thumb, held in place for 1 min, and removed from the skin. To trace and visualize the diffusion of drugs encapsulated in the microneedles, coumarin 314 (green) and SRB (red) fluorescent dyes were incorporated into PLGA and PVP matrices, respectively, as model drugs.

**Figure 5:**
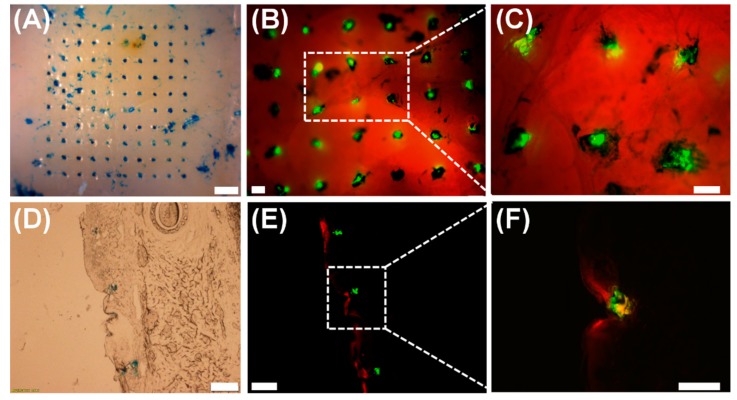
Representative images of pig cadaver skin after insertion tests. (**A**) Top view of the skin showing insertion sites. (**B**,**C**) Corresponding fluorescence images of the skin. (**D**) Cross-section of the skin. (**E**,**F**) Corresponding fluorescence images showing the dyes released in the skin at different rates. Scale bars represent 1 mm in (**A**) and 200 μm in (**B**–**F**).

Figure 5A shows a representative bright-field image of the pig cadaver skin after microneedle application. The insertion sites indicated by a blue tissue marking dye clearly demonstrate that 10 by 10 microneedles successfully penetrated the skin. The corresponding fluorescence microscopy images (Figure 5B,C) further suggest that the PLGA tips (green) remained embedded in the skin while the PVP part (red) was quickly dissolved away upon insertion. The cross-section of the insertion sites (Figure 5D) was also observed to confirm the penetration of the microneedles and to examine the diffusion behavior of model drug molecules incorporated within each layer of the microneedles. As shown in Figure 5E,F, SRB incorporated in the PVP layer spread widely at the insertion sites, whereas coumarin 314 in the PLGA layer showed limited diffusion in the skin. Our results strongly suggest that the PLGA–PVP microneedles are mechanically strong enough to pierce the skin with thumb pressure, are able to achieve reliable implantation of tips in the skin, and release multiple drugs at different rates. We anticipate that microneedles composed of multiple layers with distinct dissolution/degradation properties would be a useful way to modulate drug release rates, and desired release profiles could be obtained by adjusting various parameters, including material combinations, molecular weight, ratio of copolymers, and layer geometry and thickness.

### 3.5. In Vitro Release Profile of the Model Protein from the Layered Microneedles

**Figure 6:**
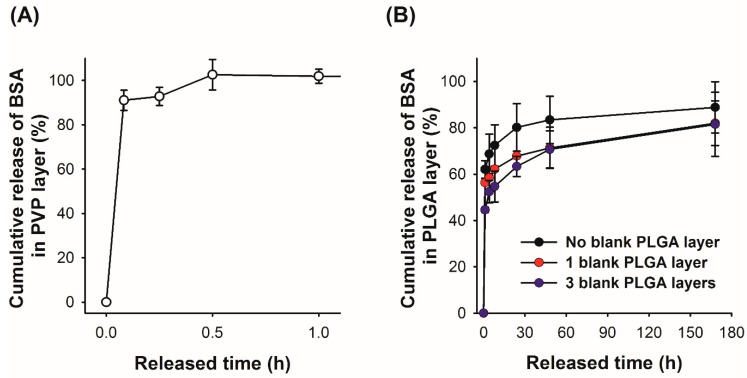
Cumulative release profile of BSA in (**A**) the PVP layer and (**B**) the PLGA layer without/with a PLGA diffusion barrier layer.

As indicated in the microneedle insertion test using the pig cadaver skin (Figure 5), the model drugs (fluorescent dyes) diffused from the layered microneedles would show a biphasic release profile. To further examine release characteristics quantitatively, we prepared two sets of PVP–PLGA layered microneedles containing BSA in either the PVP or PLGA layer, immersed the samples in PBS, and measured the amount of BSA released from each layer separately at predetermined time points. The measured BSA amount was then divided by the total amount of BSA loaded in each layer to calculate cumulative BSA release. As expected, BSA in the PVP layer was fully released within 30 min (Figure 6A) due to the quick dissolution of PVP in PBS, which is consistent with our histological observation. On the other hand, BSA in the PLGA layer showed a biphasic release profile (Figure 6B)—approximately 60% and 80% of BSA was released at 1 and 24 h, respectively, and an additional 10% was slowly released during the course of the experiments (7 days). This initial burst effect, which is frequently observed in biodegradable polymer-based drug delivery systems [44,45,46], is influenced by various factors but is mainly due to the inhomogeneous drug distribution in a polymer matrix. Specifically, for polymeric microparticle systems, the concentration of drugs in the vicinity of particle surfaces could be increased during the drying process, where drugs diffuse towards the particle surface by convection caused by solvent evaporation [44].

**Figure 7:**
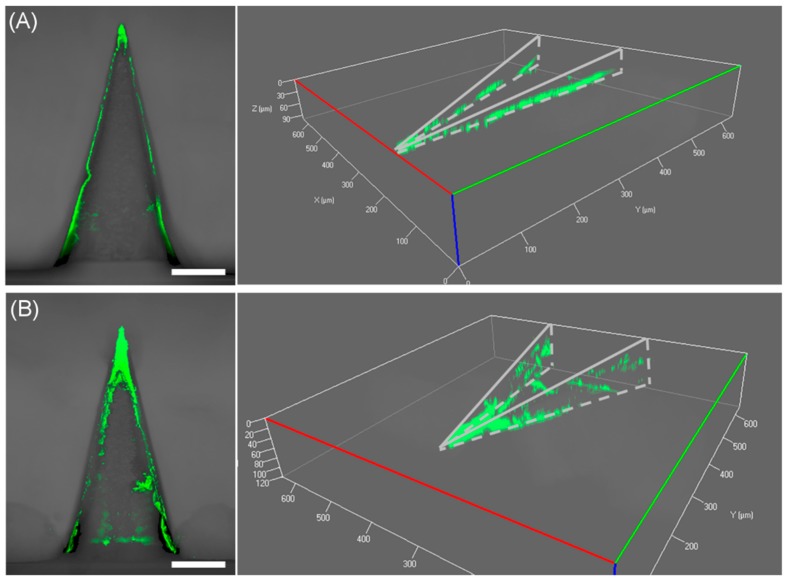
Reconstructed z-stack confocal microscopy images of (**A**) one blank layer and (**B**) three blank layers in the mold, showing the deposition profiles. Scale bars represent 100 μm.

Originally, we anticipated that microneedles formed in a mold would have a drug concentration gradient opposite to that of typical microparticle systems (i.e., low drug concentration at the microneedle surface) because the solvent will preferentially move towards the inside of the mold where the solvent–air interface exists, and thereby we could suppress the initial burst effect. However, it turned out that BSA molecules tended to be accumulated near the microneedle surface, as opposed to our expectation. We speculate that the increased surface concentration of BSA might be caused by two factors: (1) Vacuum suction from the backside of microneedle molds during spraying to fill the mold completely, which creates the airflow outwards from the mold cavity, and (2) evaporation of residual solvent after demolding of microneedles. In both cases, BSA is forced to move towards the surface of microneedles as the solvent evaporates, leading to high surface concentrations of BSA. Based on our observation, we attempted to modulate the release rate of BSA by constructing a blank PLGA layer, which could function as a physical barrier to delay the initial release of BSA. Prior to spraying a BSA-containing PLGA solution into the mold, a PLGA solution without BSA was sprayed onto the mold to form a thin outer layer of microneedles. Multilayer microneedles with blank PLGA layers of two different thicknesses were prepared by spraying the blank PLGA solution one and three times, and their release profiles were compared. As shown in Figure 6B, the addition of a blank PLGA layer was able to suppress the initial burst release of BSA, and the released BSA amounts during the first 1 h decreased as the number of blank PLGA depositions increased (approximately 56% and 44% for one and three spray depositions, respectively). This result suggests that the release of drugs from a biodegradable polymer matrix could be controlled by forming a diffusion barrier, which can be easily achieved by the proposed spray deposition method. In order to examine the deposition profile of the PLGA blank layer in the mold, the cross-section of the mold was visualized using confocal microscopy after spraying a PLGA solution containing a green fluorescent dye (coumarin 314) into the mold. As shown in Figure 7, the PLGA layer did not uniformly cover the mold surface. PLGA was mostly accumulated at the tip and was partially deposited along the edges. The coverage of mold faces was not significantly improved by increasing the number of depositions. Due to the incomplete coverage of a diffusion barrier, the initial burst release was partially suppressed, and the drug release from the faces became dominant at the later release stage as the discontinuity of the PLGA layer became larger.

## 4. Conclusions

Layered microneedles composed of biodegradable PLGA and water-soluble PVP were successfully fabricated using the spray deposition process. The fabricated microneedles possessed sufficient mechanical strength to allow reliable skin penetration. Histological examination indicated that the PLGA tips were fully embedded in the skin, while the PVP shafts were quickly dissolved away upon insertion, leading to a biphasic drug release profile. The amount of BSA in each layer was well controlled by changing either the number of spray depositions or the concentration of BSA in the spraying solution. Circular dichroism revealed a slight structural change of BSA, which might be caused by external stresses during spraying, drying, and emulsification processes. In vitro drug release studies showed the burst release from PVP and sustained release from PLGA, which was consistent with the histological observations. The release of BSA from the PLGA layer was further controlled by forming a diffusion barrier, which suppressed the initial burst to a certain degree. We anticipate that fine-tuning of drug release profiles could be achievable by optimizing process parameters, such as initial drug concentration in each layer and the number of depositions, and by controlling the thickness and uniformity of the diffusion barrier. Moreover, an extended study including thermal analysis, such as differential scanning calorimetry, would help elucidate the influence of process parameters and materials choice on tailoring drug release profiles. Also, it is highly desirable to perform an animal study to correlate in vitro drug release and in vivo drug absorption. In conclusion, the layered microneedles could be a simple yet effective approach to modulate drug release profiles and would be useful to deliver multiple drugs or vaccines in a controlled manner.

## Figures and Tables

**Table 1:** Structural stability of BSA in PLGA and PVP layers. The distributions of secondary structure were estimated from circular dichroism (CD) spectra, and the changes in the tertiary structure were estimated by comparing the peak fluorescence intensity of samples with that of native BSA at 339 nm.

Group	Secondary Structure (%)				Tertiary Structure (%)
	α-Helix	β-Sheet	Turn	Unordered	
Native BSA	62.5 ± 1.5	7.3 ± 0.6	10.7 ± 2.3	19.7 ± 4.9	100.0 ± 3.2
Degraded BSA	44.0 * ± 5.2	20.0 * ± 3.2	14.1 * ± 1.7	22.2 * ± 2.5	51.3 * ± 1.4
BSA in PLGA layer	54.2 * ± 3.1	8.9 * ± 1.4	13.3 * ± 1.2	23.7 * ± 0.6	93.3 * ± 8.1
BSA in PVP layer	56.3 * ± 1.6	9.4 *± 1.5	11.0 ± 2.3	23.3 * ± 5.1	99.2 ± 4.5

Asterisk (*) indicates significant differences as compared with native BSA (n = 6, *p* < 0.05).

## Supplementary Materials

The following are available online at http://www.mdpi.com/2073-4360/11/2/369/s1, Figure S1: (A) CD spectra of native, degraded BSA, and BSA in PLGA and PVP layer. (B) Fluorescence spectra of native, degraded BSA, and BSA in PLGA and PVP layer.
