# Supplementary material for: Spray-Formed Layered Polymer Microneedles for Controlled Biphasic Drug Delivery
Source: Polymers (Basel). 2019 Feb 20;11(2):369. doi: 10.3390/polym11020369 (PMC6419157; doi:10.3390/polym11020369)
Supplement: Supplementary file 1 [file polymers-11-00369-s001.pdf]

Article

Spray-formed layered polymer microneedles for controlled biphasic drug delivery

Seok Chan Park[1, 2§], Min Jung Kim[1, 2§], Seung-Ki Baek[3], Jung-Hwan Park[4, 5], Seong-O Choi[1, 2*]

1 Nanotechnology Innovation Center of Kansas State, Kansas State University, Manhattan, KS, USA; mj1217@ksu.edu; schpark@ksu.edu; sochoi@ksu.edu

2 Department of Anatomy and Physiology, College of Veterinary Medicine, Kansas State University, Manhattan, KS, USA

3 QuadMedicine R&D Centre, QuadMedicine Inc., Seongnam, Republic of Korea; bsk@quadmedicine.com

4 Department of BioNano Technology, College of BioNano Technology, Gachon University, Seongnam, Republic of Korea; pa90201@gachon.ac.kr

5 Gachon BioNano Research Institute, Gachon University, Seongnam, Republic of Korea

* Correspondence: sochoi@ksu.edu

§ These authors contributed equally to this work.

Supplementary Materials

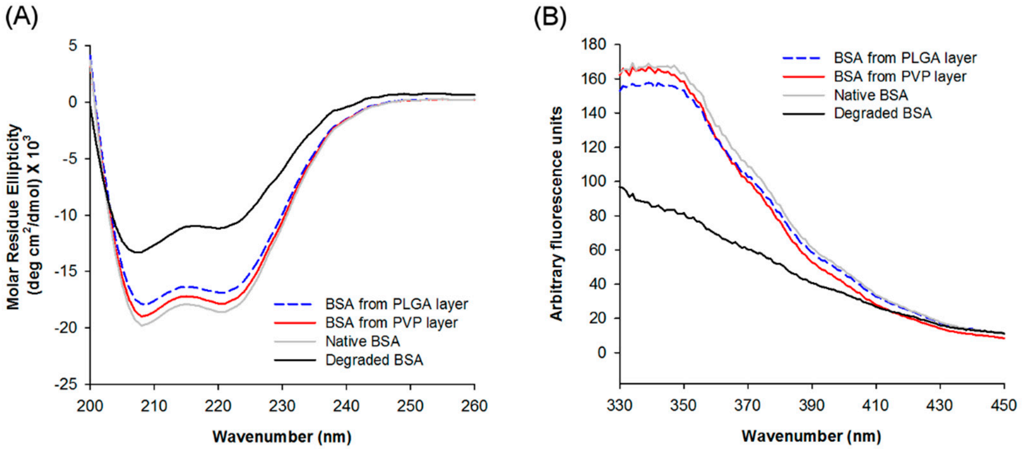

Figure S1. (A) CD spectra of native, degraded BSA, and BSA in PLGA and PVP layer. (B) Fluorescence spectra of native, degraded BSA, and BSA in PLGA and PVP layer.

© 2019 by the authors. Submitted for possible open access publication under the terms and conditions of the Creative Commons Attribution (CC BY) license (http://creativecommons.org/licenses/by/4.0/).
